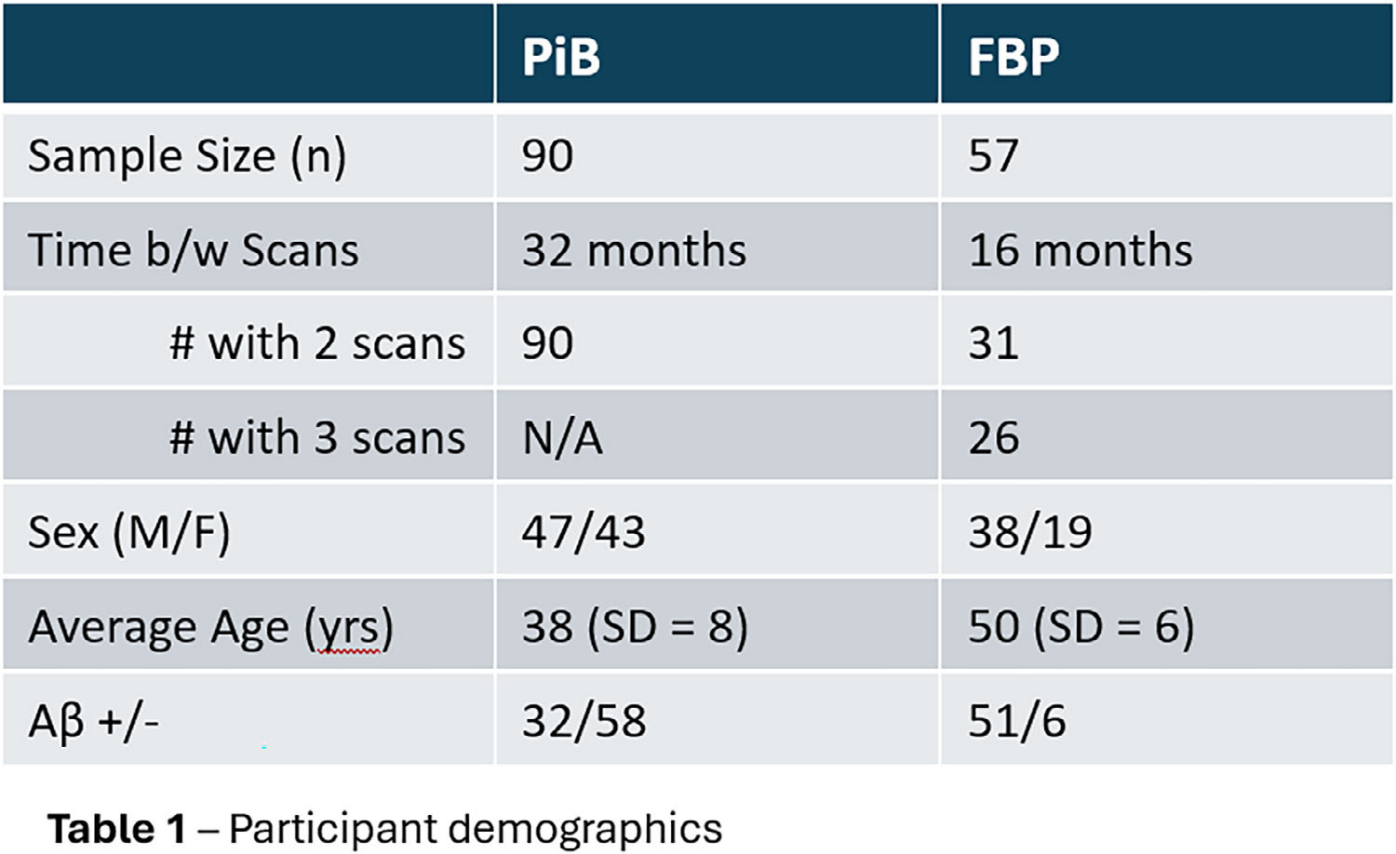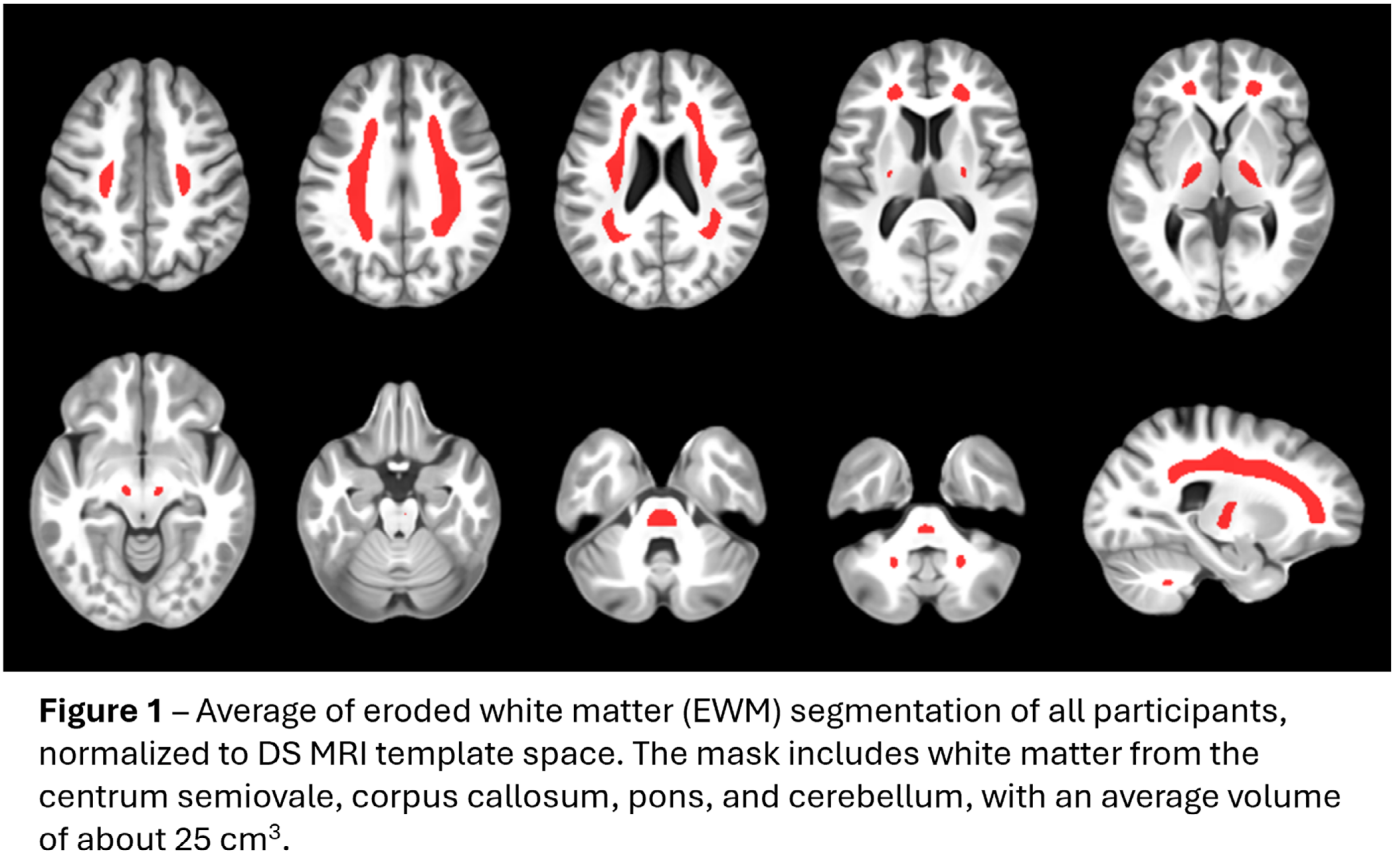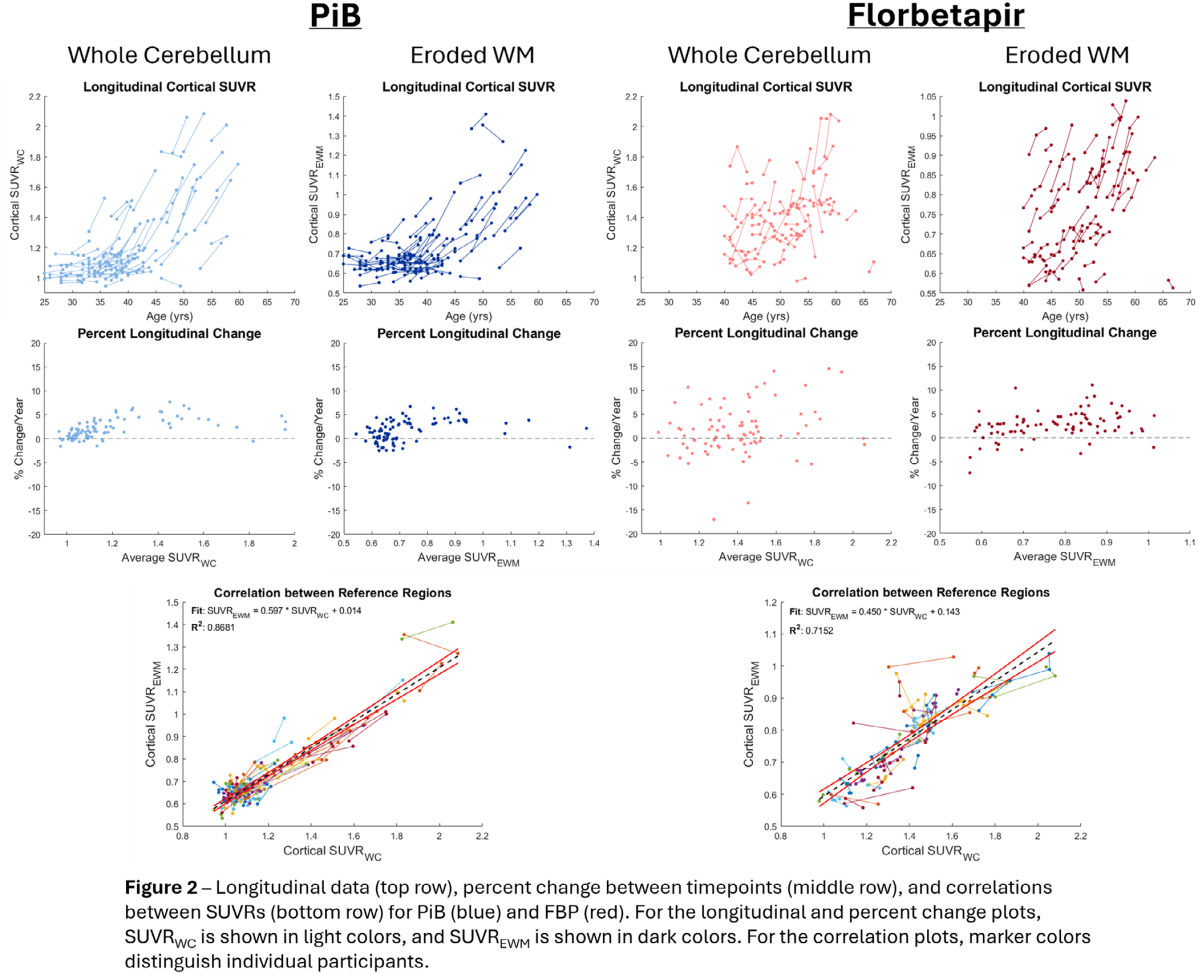# Comparison of reference region stability for longitudinal amyloid PET in Down syndrome

**DOI:** 10.1002/alz.092918

**Published:** 2025-01-09

**Authors:** Max McLachlan, Jeremy P. Rouanet, Arun Garimella, Julie C Price, Dana Tudorascu, Charles M Laymon, David B. Keator, Tobey J. Betthauser, William Charles Kreisl, Patrick J. Lao, Davneet S Minhas, William E Klunk, Annie Cohen, Benjamin L Handen, Tim D Fryer, Shahid Zaman, Robert A. Koeppe, Elizabeth Head, Mark Mapstone, Brecca Bettcher, Lisette LeMerise, Andrew K McVea, Alexandra H DiFilippo, Matthew D Zammit, Sigan L Hartley, Bradley T. Christian

**Affiliations:** ^1^ Waisman Center, University of Wisconsin‐Madison, Madison, WI USA; ^2^ University of California, Irvine, Irvine, CA USA; ^3^ Massachusetts General Hospital, Boston, MA USA; ^4^ University of Pittsburgh, Pittsburgh, PA USA; ^5^ University of Wisconsin School of Medicine and Public Health, Madison, WI USA; ^6^ Columbia University Irving Medical Center, New York, NY USA; ^7^ University of Cambridge, Cambridge UK; ^8^ University of Michigan, Ann Arbor, MI USA

## Abstract

**Background:**

The cerebellum is frequently used as the reference region for amyloid PET analysis. However, this reference region has been shown to demonstrate longitudinal variability, particularly with [^18^F]florbetapir (FBP) PET (Landau, JNM 2015). For investigations in individuals with Down syndrome (DS), cerebellar atrophy and rapid disease progression may increase these longitudinal variabilities. Although white matter possesses different non‐displaceable uptake properties, the relative lack of specific binding makes white matter a suitable reference region for longitudinal studies. This work compares the observed longitudinal change when using whole cerebellum and white matter reference regions in [^18^F]FBP and [^11^C]PiB scans of adults with DS.

**Method:**

Participants with DS, recruited through the ABC‐DS study, underwent longitudinal PiB or FBP PET imaging and T1w MRIs (Table 1 lists cohort differences). PET images were smoothed to 8mm resolution, summed 50‐70 min, co‐registered with the MRI, and normalized to a common DS MRI template (LeMerise, 2022). GAINN whole cerebellum (WC) VOI was applied to create SUVR_WC_. Whole brain white matter was segmented in native space using SPM, smoothed to PET resolution, and eroded to 90% tissue probability. The resulting eroded white matter (EWM) mask was used as reference to create SUVR_EWM_. Average SUVR was calculated for GAINN global cortex (CTX). Longitudinal scans were assessed for correlations between reference region strategies and average rate of SUVR change: % Change/year = (SUVR_2_‐SUVR_1_)/(SUVR_1_*Δt).

**Result:**

Figure 1 displays the averaged EWM reference template. Figure 2 displays longitudinal PET data and regressions between SUVRs. Across participants, SUVR_WC_ shows 78/90 (PiB) and 50/83 (FBP) between‐scan increases. SUVR_EWM_ shows 66/90 (PiB) and 71/83 (FBP) between‐scan increases. For A+ individuals (18CL cutoff), the average difference (% Change_EWM_ ‐ % Change_WC_)/year is ‐0.5%/year [‐1.2,0.3] (PiB) and 1.9%/yr [0.5,3.3]** (FBP). FBP group SD in % Change/year decreases from 5.6% (WC) to 2.9% (EWM).

**Conclusion:**

As observed in LOAD, SUVR_EWM_ demonstrates lower group variability and greater longitudinal change in FBP. SUVR_EWM_ shows strong agreement with SUVR_WC_ in PiB. These data suggest that an EWM reference region can reduce variability in longitudinal FBP studies in DS.